# Exploring the Relationships between Four New Species of Boletoid Fungi from Northern China and Their Related Species

**DOI:** 10.3390/jof8030218

**Published:** 2022-02-22

**Authors:** Yang Wang, Yong-Lan Tuo, Dong-Mei Wu, Neng Gao, Zhen-Hao Zhang, Gu Rao, Xiao-Min Wang, Jing Wang, Dan Dai, Yu Li, Bo Zhang

**Affiliations:** 1 College of Plant Protection, Shenyang Agricultural University, Shenyang 110866, China; lesireyang@163.com; 2 Engineering Research Center of Chinese Ministry of Education for Edible and Medicinal Fungi, Jilin Agricultural University, Changchun 130118, China; tuoyonglan66@163.com (Y.-L.T.); zzhzz34@163.com (Z.-H.Z.); raogufungi@163.com (G.R.); 3 Biotechnology Research Institute, Xinjiang Academy of Agricultural and Reclamation Sciences, Shihezi 830011, China; wdm0999123@sina.com (D.-M.W.); gaoneng520@163.com (N.G.); 4 Institute of Soil and Fertilizer, Guizhou Academy of Agricultural Sciences, Guiyang 550006, China; rdl1916@163.com; 5 Institute of Biology, Guizhou Academy of Sciences, Guiyang 550001, China; 17687190411@163.com; 6 Institute of Agricultural Applied Microbiology, Jiangxi Academy of Agricultural Sciences, Nanchang 330200, China; m13082447311@163.com

**Keywords:** *Boletales*, biodiversity, molecular analyses, taxonomy

## Abstract

The family Boletaceae primarily represents ectomycorrhizal fungi, which play an essential ecological role in forest ecosystems. Although the Boletaceae family has been subject to a relatively global and comprehensive history of work, novel species and genera are continually described. During this investigation in northern China, many specimens of boletoid fungi were collected. Based on the study of their morphology and phylogeny, four new species, *Butyriboletus pseudoroseoflavus*, *Butyriboletus subregius*, *Tengioboletus subglutinosus*, and *Suillellus lacrymibasidiatus*, are introduced. Morphological evidence and phylogenetic analyses of the single or combined dataset (ITS or 28S, *rpb*1, *rpb*2, and *tef*1) confirmed these to be four new species. The evidence and analyses indicated the new species’ relationships with other species within their genera. Detailed descriptions, color photographs, and line drawings are provided. The species of *Butyriboletus* in China were compared in detail and the worldwide keys of *Tengioboletus* and *Suillellus* were given.

## 1. Introduction

Boletaceae Chevall. [1], a family with more than 70 genera, is one of the most prominent and diverse among the basidiomycetes [2]. It is mainly characterized by being tubulose with infrequent lamellate or loculate hymenophora, and by a fleshy context. Most Boletaceae species have value for humans and are essential for mutualistic symbiosis with trees [3,4,5,6]. Although the family Boletaceae was established nearly two centuries ago, the species diversity of the family increased significantly in the last few decades [7,8,9,10,11,12,13,14,15,16,17,18,19]. Because the morphology of Boletaceae has convergent characteristics, the classification did not correspond to the phylogeny of Boletaceae for a long time. With the development of molecular biology, the method of genealogical concordance phylogenetic species recognition (GCPSR) [20] was used to identify species of fungi, resolved some doubts about the status of taxa, and contributed to a better understanding of the relationships of the genera in this family [5,21,22]. In the past two decades, new genera and new species have rapidly increased, and the evolution of ectomycorrhizas of Boletales was gradually disclosed [23,24].

In China, the family Boletaceae has continued to receive increasing attention from mycologists [5,25,26,27,28,29,30,31,32]. However, the previous studies were focused on southern China, and the species diversity remained unclear in northern China. During previous field collection in the north of China, we obtained many specimens. Based on our analyses of their morphology and phylogeny, we propose four new species: *Butyriboletus pseudoroseoflavus*, *Butyriboletus subregius*, *Tengioboletus subglutinosus*, and *Suillellus lacrymibasidiatus*.

*Butyriboletus* was erected by Arora et al. [33] to accommodate the *Boletus* sect. *Appendiculati*. It is characterized by a reddish to brown pileus and a yellow hymenophore, usually staining blue when bruised. Five species have been described in China, i.e., *Bu*. *huangnianlaii* N.K. Zeng, H. Chai & Zhi Q. Liang [28], *Bu*. *pseudospeciosus* Kuan Zhao & Zhu L. Yang [5], *Bu*. *roseoflavus* (Hai B. Li & Hai L. Wei), D. Arora & J.L. Frank [33], *Bu*. *sanicibus* D. Arora & J.L. Frank [33], and *Bu*. *yicibus* D. Arora & J.L. Frank [32].

*Tengioboletus* was established by Wu et al. [5], including three species: *T*. *glutinosus* G. Wu & Zhu L. Yang, *T*. *reticulatus* G. Wu & Zhu L. Yang, and *T*. *fujianensis* N.K. Zeng & Zhi Q. Liang [5,34]. *Tengioboletus* can be distinguished easily from other Boletaceae genera by combining the following characteristics: a yellow context; hymenophore that change color when injured; tubes that are concolorous with the surface; cystidia that are scattered; subfusiform-ventricose or clavate shape; and an epithelium to ixotrichodermium pileipellis.

*Suillellus*, typified by *Boletus luridus* Schaeff, was established by Murrill in 1909 [7]. According to Vizzini et al. [13], *Suillellus* s.str. is characterized by basidiomata that are usually slender, stipes that are cylindrical and sometimes covered with reticulation, pileus that are reddish brown to olivaceous and turn to blue when bruised, the presence or absence of Bataille’s line, and a context that is reddish in the stipe base and bluing when exposed to air and positive amyloid reaction.

## 2. Materials and Methods

### 2.1. Samplings and Morphological Analyses

Materials were collected from Jilin province and the Xinjiang Uygur Autonomous Region, China. Voucher specimens were deposited in the Herbarium of Mycology of the Jilin Agriculture University (HMJAU). Descriptions of the colors of basidiomata used color coding from Kornerup and Wanscher [35]. The micro-morphological structures were performed in a 5% KOH solution and then in a 1% Congo Red or Melzer’s reagent solution. The amyloid reaction was tested following Imler’s procedure [36,37]. The abbreviations for basidiospore measurements (n/m/p) indicate “n” basidiospores from “m” basidiomata of “p” specimens. The sizes of basidiospores are given as (a) b–m–c (d), where “a” is the smallest value, “d” is the largest value, “m” is the average value point, and “b–c” covers a minimum of 95% of the values. “Q” stands for the ratio of the length and the width of the basidiospores and “Q ± av” stands for for the average Q of all basidiospores ± sample standard deviation. The scanning electron microscope (SEM) was used to observe the ultrastructure of the spores.

### 2.2. DNA Extraction, PCR Amplification, and Sequencing

Genomic DNA was extracted from dried specimens, using the NuClean Plant Genomic DNA kit (CWBIO). For the amplification of ITS, 28S, *rpb*1, *rpb*2, and *tef*1, we used primer pairs ITS1/4, LROR/LR5, RPB1-B-F/RPB1-B-R, RPB2-B-F1/RPB2-B-R, and 983F/1567R, respectively [5,22,25,38,39,40,41,42]. PCR amplification procedures were set to refer to Zhang et al. [43], White et al. [39], and Kuo and Ortiz-Santana [44]. Then, PCR productions were sent to Sangon Biotech Co. Ltd. (Shanghai, China) to be directly sequenced using the ABI 3730xl DNA analyzer.

### 2.3. Data Analysis

Newly generated sequences were uploaded to NCBI (https://www.ncbi.nlm.nih.gov/, accessed on 10 January 2022), as shown in Table 1, with other similar sequences downloaded from the NCBI and UNITE (https://unite.ut.ee/, accessed on 10 January 2022) datasets. DNA sequences were aligned and manually modified using Bioedit v7.1.3 [45]. In the multi-locus dataset (28S + *rpb*1 + *rpb*2 + *tef*1) of *Tengioboletus*, 894 bp for 28S, 758 bp for *rpb*1, 710 bp for *rpb*2, and 638 bp for *tef*1, and in the four-locus dataset (*tef*1 + 28S + *rpb*2 + ITS) of *Butyriboletus*, 730 bp for *tef*1, 863 bp for 28S, 834 bp for *rpb*2, and 809 bp for ITS. The data used for phylogenetic analyses for *Suillellus* included the ITS dataset and a multi-locus dataset (*tef*1 + 28S + *rpb*1 + *rpb*2), For the multi-locus dataset, 907 bp for 28S, 791 bp for *rpb*1, 719 bp for *rpb*2, and 631 bp for *tef*1. The best models of the multi-locus datasets were searched via PartitionFinder 2 [46]. Meanwhile, the best model of the ITS dataset was searched via Modelfinder [47]. Phylogenetic analyses were carried out using the maximum likelihood method (ML) and the Bayes inference (BI) method. The models employed for each of the four loci of *Tengioboletus*, and *Butyriboletus* were GTR + I + G for 28S, SYM + G for *rpb*1, K80 + G for *rpb*2, SYM + I + G for *tef*1, and GTR + I + G for ITS, GTR + I + G for 28S, K80 + G for *rpb*2, SYM + I + G for *tef*1, respectively. For the multi-locus dataset of *Suillellus*, the best models for each locus were K80 + I + G for *rpb*1 and SYM + I + G for 28S, *rpb*2, and *tef*1. In the ITS dataset of *Suillellus*, the best models for ML analysis and BI analyses were K2P + I + G4. For ML analyses, the datasets were analyzed using IQ-TREE [48] under an ultrafast bootstrap, with 5000 replicates. For BI analyses, the multi-locus datasets were analyzed using MrBayes 3.2.6 [49], running in a total of 2,000,000 generations, and sampled every 1000 generations. The initial 25% of the sampled data were discarded as burn-in. Other parameters were kept at their default settings.

## 3. Results

### 3.1. Molecular Phylogeny

**Figure 1:**
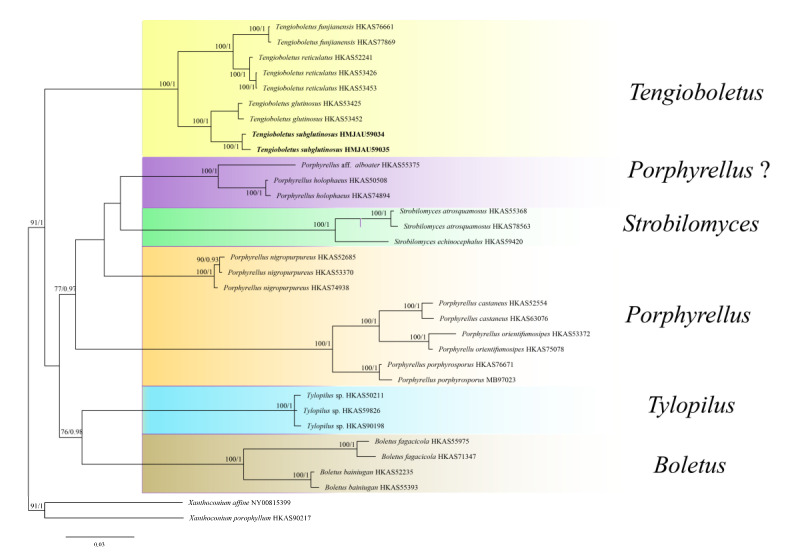
Phylogenetic analysis of *Tengioboletus* inferred from ML analysis. BP value (>70) and PP value (>8) are shown around branches. Our new species sequences are indicated in bold.

The four-locus dataset (28S + *rpb*1 + *rpb*2 + *tef*1) of *Tengioboletus* (Supplementary File S1) contained 33 sequences and 3000 bp nucleotide sites. The alignment was submitted to TreeBASE (http://purl.org/phylo/treebase/phylows/study/TB2:S29030, accessed on 15 January 2022). Because the ML tree’s topology was the same as the BI tree’s topology, only the ML tree was shown (Figure 1). *Xanthoconium affine* (Peck) Singer and *Xanthoconium porophyllum* G. Wu & Zhu L. Yang were chosen as outgroups. The phylogenetic tree showed that two *T. subglutinosus* sequences formed an independent lineage, with bootstrap proportions (BP) = 100 and posterior probability (PP) = 1, and formed a sister group with *T. glutinosus* (BP = 100, PP = 1).

**Figure 2:**
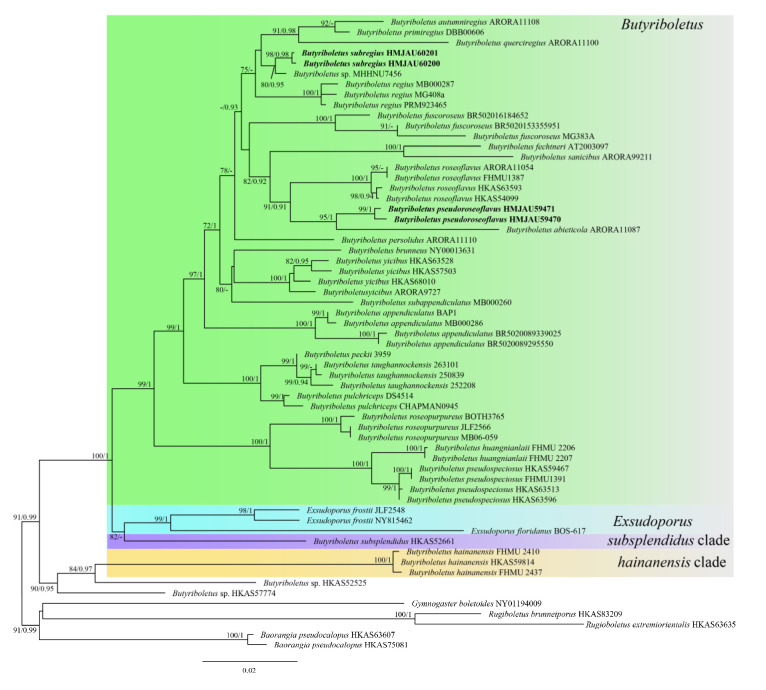
Phylogenetic analysis of *Butyriboletus* inferred from ML analysis. BP value (>70) and PP value (>9) are shown around branches. Our new species sequences are indicated in bold.

The four-locus dataset (ITS + 28S + *tef*1 + *rpb*2) of *Butyriboletus* (Supplementary File S2) consisted of 58 taxa and 3011 nucleotide sites (Figure 2). The alignment was submitted to TreeBASE (http://purl.org/phylo/treebase/phylows/study/TB2:S29034, accessed on 15 January 2022). *Baorangia pseudocalopus* (Hongo) G. Wu & Zhu L. Yang was chosen as the outgroup. The phylogram indicated our collections—HMJAU59471, HMJAU59470, and HMJAU60200, HMJAU60201—were grouped together respectively and formed two independent lineages with high support value (BP = 100, PP = 1 and BP = 99, PP = 1).

**Figure 3:**
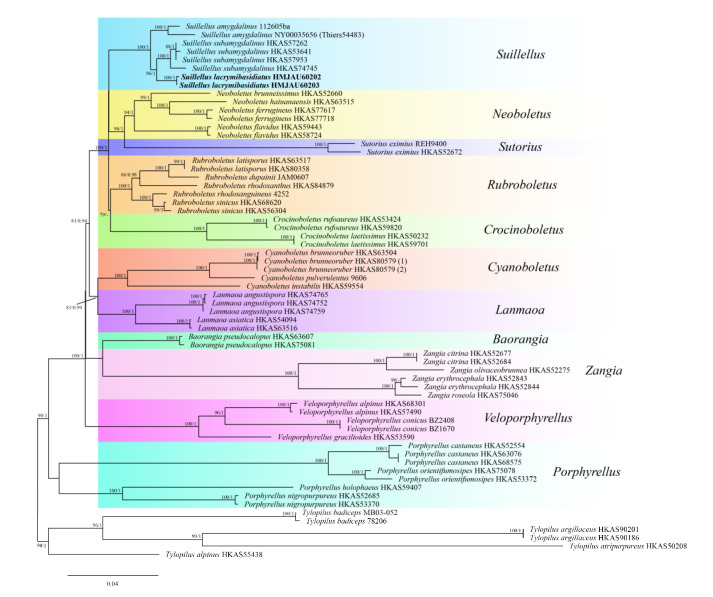
Phylogenetic analysis of *Suillellus* inferred from ML analysis based on the multi-locus dataset. BP value (>70) and PP value (>9) are shown around branches. Our new species sequences are indicated in bold.

**Figure 4:**
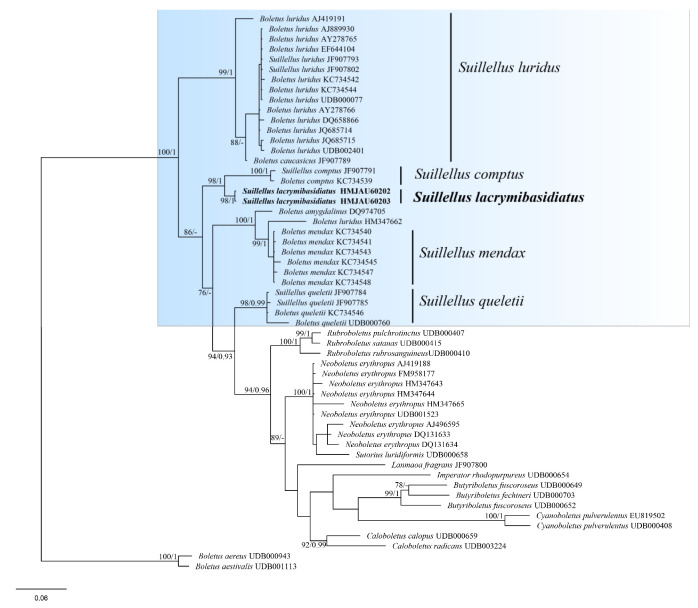
Phylogenetic analysis of *Suillellus* inferred from Bayes inference analysis based on ITS dataset. BP value (>70) and PP value (>9) are shown around branches. Our new species sequences are indicated in bold.

The four-locus dataset (28S + *rpb*1 + *rpb*2 + *tef*1) of *Suillellus* (Supplementary File S3) involved 64 taxa and 3048 bp sites. *Tylopilus alpinus* Y.C. Li & Zhu L. Yang and *Tylopilus atripurpureus* (Corner) E. Horak were selected as outgroups. The alignment was submitted to TreeBASE (http://purl.org/phylo/treebase/phylows/study/TB2:S29037, accessed on 15 January 2022). The phylogram showed our species belongs to *Suillellus* (Figure 3). It formed an independent sister clade to *Suillellus subamygdalinus* Kuan Zhao & Zhu L. Yang, with a solid support (BP = 92, PP = 1). The ITS dataset of *Suillellus* (Supplementary File S4) consisted of 55 taxa and 885 bp sites. *Boletus aestivalis* (Paulet) Fr. and *Boletus aereus* Bull. were chosen as outgroups (Figure 4). The alignment was submitted to TreeBASE (http://purl.org/phylo/treebase/phylows/study/TB2:S29087, accessed on 15 January 2022). The phylogram showed that our species was close to *Suillellus comptus* (Simonini) Vizzini, Simonini & Gelardi and formed an independent and robust support clade (BP = 98, PP = 1).

### 3.2. Taxonomy

*Butyriboletus pseudoroseoflavus* Yang Wang, Bo Zhang & Yu Li, sp. nov.

Mycobank No.: 842167.

**Figure 5:**
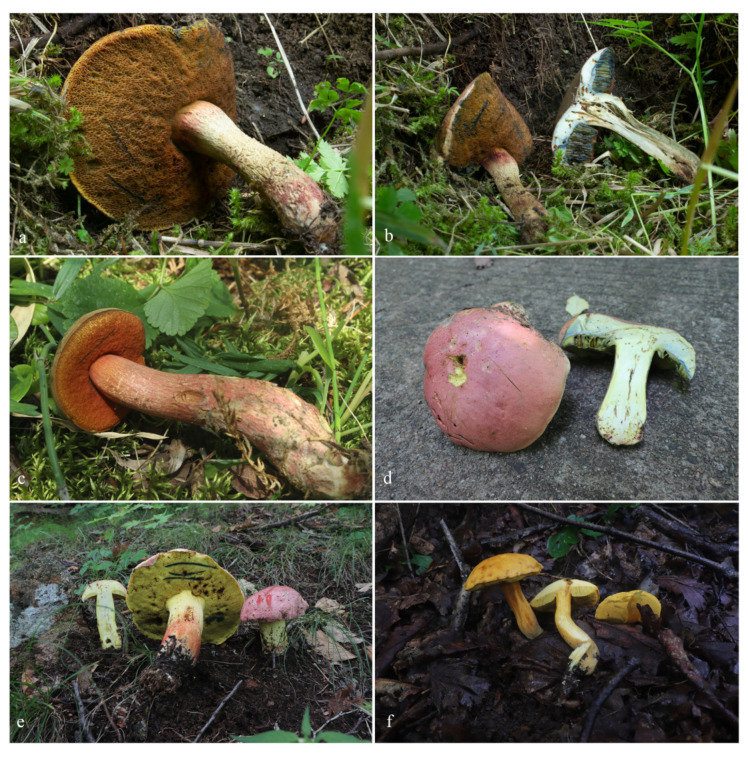
Basidiomata of boletes. (**a**–**c**) *Suillellus lacrymibasidiatus*; (**d**) *Butyriboletus subregius*; (**e**) *Butyriboletus pseudoroseoflavus* (e from HMJAU 59470); (**f**) *Tengioboletus subglutinosus* (f from HMJAU 59037). (**a**–**c**) Photos by Yang Wang; (**d**–**f**) Photos by Yong-Lan Tuo.

**Figure 6:**
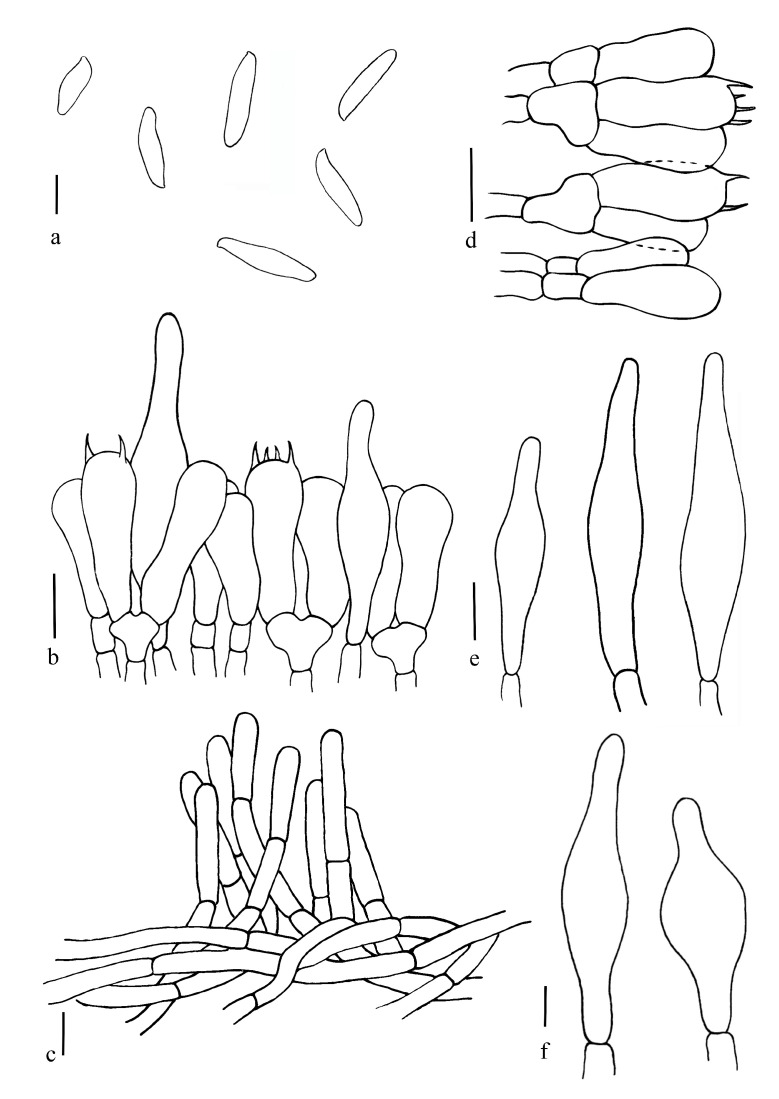
*Butyriboletus pseudoroseoflavus*. (**a**) Basidiospores; (**b**) Basidia and pleurocystidia; (**c**) Pileipellis; (**d**) Stipitipellis; (**e**) Pleurocystidia; (**f**) Cheilocystidia. Scale bars: (**b**–**e**) =10 μm; (**a**,**f**) =5 μm.

**Figure 7:**
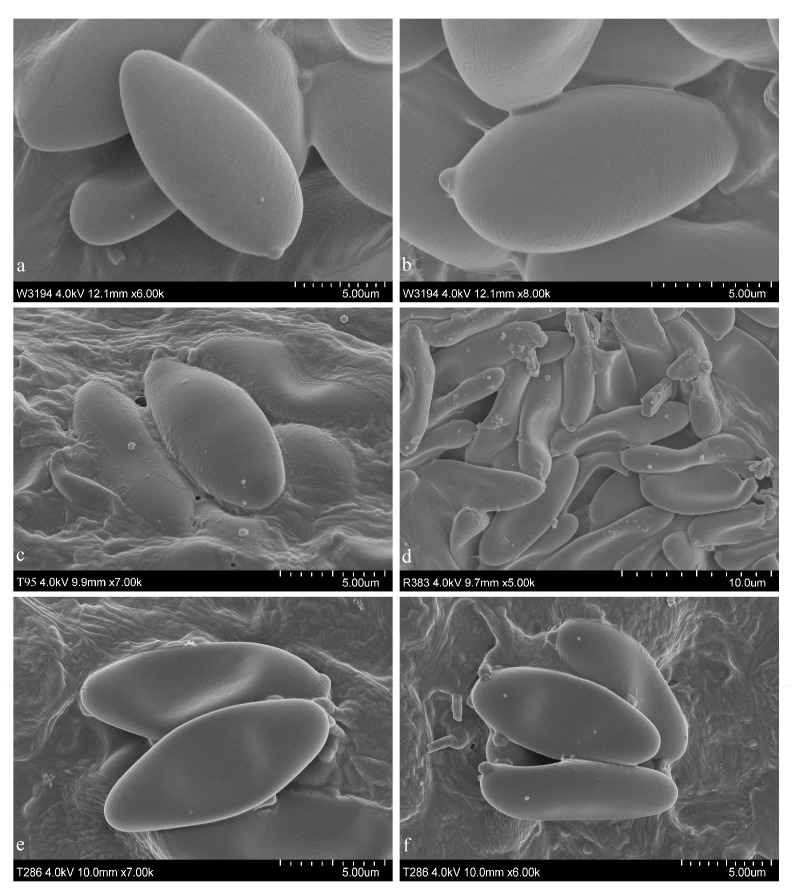
Basidiospores observed in the SEM. (**a**,**b**) *Suillellus lacrymibasidiatus*; (**c**) *Butyriboletus subregius*; (**d**) *Butyriboletus pseudoroseoflavus*; (**e**,**f**) *Tengioboletus subglutinosus*.

Figure 5e, Figure 6 and Figure 7d.

Etymology. The epithet “*pseudoroseoflavus*” refers to its similarity to *B. roseoflavus*.

Holotypus. China. Jilin Province, Jian city, Wunvfeng National Forest Park, 125°34′33″ E, 40°52′7″ N, under *Quercus mongolica*, on dark-brown soil, alt. 1210 m, 16 August 2019, Gu Rao 383 (HMJAU 59471!).

Basidiomata large. Pileus 13.5–17.0 cm in diameter, hemispherical to applanate, with slightly or distinctly appendiculate margin, sometimes incurved at the margin when young; surface tomentose, pink (12A4) to greyish rose (11B5), context 1.1–1.8 cm thick, light yellow (1A5), unchanging in color when injured. Hymenophore adnate to decurrent, surface greenish yellow (1B8), becoming greenish blue (23B8) quickly when bruised; pores round to angular, ca. 1–3/mm; tubes 1.5–1.7 cm long, concolorous with pore surface, unchanging color when injured. Stipe 9.0–14.7 × 2.0–3.6 cm, subcylindrical, robust, yellow on the upper portion, vivid red (10A8) downwards, surface almost wholly covered yellow (2B8) reticulation or at least upper two thirds; basal mycelium white.

Basidiospores (60/3/2) (7.0) 10.2–10.6–11.0 (16.0) × (2.0) 3.1–3.2–3.7 (4.0) μm, Q = (2.0) 2.5–4.6 (5.3), Qm = 3.30 ± 0.58, elongate oblong to subfusoid, inequilateral with a suprahilar depression in side view, light yellow in 5% KOH, smooth. Hymenophoral trama boletoid, hyphae cylindrical, 2.5–10 μm wide. Basidia clavate, thin-walled, 16.0–33.0 × 2.0–10.0 μm, 2- and 4-spored. Cheilocystidia 31.5–50.0 × 5.0–10.0 μm, narrowly lageniform, thin-walled, pale yellow in 5% KOH. Pleurocystidia 37.5–62.5 × 5.0–11.5 μm, similar to cheilocystidia in shape. Pileipellis trichodermium, filamentous hyphae 1.7–7.5 μm wide. Stipitipellis fertile, hymeniform with thin-walled and inflated terminal cells (13.8–26.0 × 6.8–13.8 μm). Stipe trama composed of parallel hyphae 2.5–7.5 μm wide. Clamp connections not observed.

Habitat: solitary or scattered on a dark-brown soil of *Quercus mongolica* forest.

Known distribution: currently, only known from Jilin province, China.

Additional collection examined: China. Jilin Province, Jian city, Wunvfeng National Forest Park, 125°34′33″ E, 40°52′7″ N, under *Quercus mongolica*, on dark-brown soil, alt. 950 m, 5 August 2020, Yong-Lan Tuo 274 (HMJAU 59470).

Notes: *Butyriboletus pseudoroseoflavus* is characterized by a pink to greyish rose pileus, greenish yellow pores changing to greenish blue when bruised, a stipe surface almost wholly covered with yellow reticulation, a stipe of unchanging color when injured, and large and narrow basidiospores. Morphologically and phylogenetically, *Bu. pseudoroseoflavus* is similar to *Bu. roseoflavus* (Hai B. Li & Hai L. Wei) D. Arora & J.L. Frank, which was initially described in specimens from eastern China (Zhejiang province) and southwestern China (Yunnan province) by Li et al. [67]. However, *Bu. pseudoroseoflavus* differs from *Bu. roseoflavus* in its adnate to decurrent hymenophore and its relatively larger and narrower basidiospores, with a more considerable Q value and pleurocystidia larger than cheilocystidia [5]. In morphological features, *Bu. pseudoroseoflavus* is also similar to *Bu. cepaeodoratus* (Taneyama & Har. Takah.) Vizzini & Gelardi, *Bu. roseogriseus* (Šutara, M. Graca, M. Kolařík, Janda & Kříž) Vizzini & Gelardi, *Bu. primiregius* D. Arora & J.L. Frank, *Bu. regius* (Krombh.) D. Arora & J.L. Frank., and Bu. fuscoroseus (Smotl.) Vizzini & Gelardi, but the pileus of *Bu. cepaeodoratus* always has a duller color, its stipe stains blue when injured, and its basidiospores are broader than those of *Bu. pseudoroseoflavus* [68]. Both stipe and context of *Bu. roseogriseus* and *Bu. primiregius* turn blue when injured, and have broader basidiospores, Q = (1.95) 2.20–2.42 (2.57) and Q = 3.5, respectively [32,56]. The pores of *Bu. regius* are unchanging to blue when bruised; the stipe is usually ventricose when young, showing at the base rare faintly reddish or purplish spots, with basidiospores weakly dextrinoid [69]. *Butyriboletus fuscoroseus* is characterized by its brown-pink, reddish brown, or purplish brown pileus, decurrent hymenophore, stipe staining blue when bruised or cut, and the narrow basidiospores [56]. Phylogenetically, *Bu. pseudoroseoflavus* is similar to *Bu. abieticola*. However, *Bu. abieticola* is characterized by a light rose-colored pileus, with tan-colored spots interspersed, a white context, a hymenium dextrinoid, and hyaline spiral incrustations on most hyphae [70]. Reference Table 2 provides the critical characteristics distinguishing *Bu. pseudoroseoflavus* from other species in China.

*Butyriboletus subregius* Yang Wang, Bo Zhang & Yu Li, sp. nov.

Mycobank No.: 842517.

**Figure 8:**
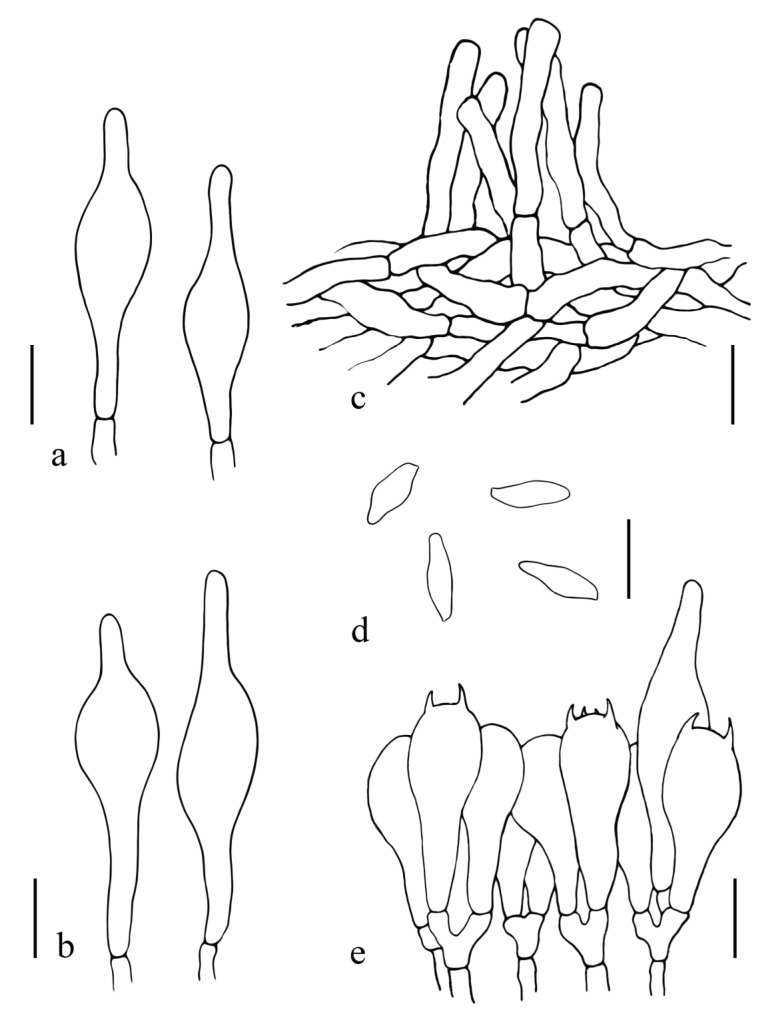
*Butyriboletus subregius*. (**a**) Pleurocystidia; (**b**) Cheilocystidia; (**c**) Pileipellis; (**d**) Basidiospores. (**e**) Pleurocystidia and basidia. Scale bars: 10 μm.

Figure 5d, Figure 7c and Figure 8.

Etymology.:“sub” means “near,” named because it is similar to *B. regius*.

Holotypus: China. Jilin Province, Jian city, Wunvfeng National Forest Park, 125°34′33″ E, 40°52′7″ N, under *Quercus mongolica*, on dark-brown soil, alt. 1050 m, 7 July 2020, Yong-Lan Tuo 95 (HMJAU 60200!).

Basidiomata middle to large. Pileus 7.0–13.0 cm in diameter, hemispherical or broadly hemispherical at maturity, with distinctly appendiculate margin initially, surface dry, covered with weak or distinct tomentum, pastel pink (11A4–5), context yellowish green (30A6), turning blue when cut. Hymenophore weakly decurrent, covered with a layer of whitish mycelium (1A1) when young, surface yellowish green (29A6), bluing when bruised, pores angular to nearly round, ca. 4–5/mm; tubes concolorous with hymenophore surface, about 1.1 cm long, turning blue when cut. Stipe 11.0–14.5 × 4.4–5.0 cm, subcylindrical or enlarged downwards, yellowish green (29A6) at maturity, covered with pastel red stains when young, upper 2/3 portion covered with yellowish green (29A6) reticulation, staining blue when bruised, context yellowish green (30A6), changing weakly to blue when cut.

Basidiospores (60/2/2) (10.0) 11.1–11.3–11.5 (13.0) × (3.0) 4.0–4.1–4.2 (5.0) μm, Q = (2.22) 2.40–3.00 (4.00), Qm = 2.76 ± 0.31, subfusoid to subcylindrical, inequilateral with a suprahilar depression in side view, brownish yellow in 5% KOH, smooth. Basidia 21.0–36.0 × 8.0–12.5 μm, clavate, 2– and 4–spored. Hymenophoral trama boletoid, composed of hyphae 4–7 μm in diameter. Pleurocystidia 36.3–56.7 × 7.0–14.6 μm, narrowly lageniform, thin-walled, yellowish in 5% KOH. Cheilocystidia 22.0–50.5 × 5.5–12.4 μm, narrowly lageniform. Pileipellis a trichodermium, composed of filamentous hyphae, 3.0–6.5 μm wide. Stipitipellis fertile, hymeniform, caulocystidia 23.0–43.5 × 9.0–12.5 μm, narrowly lageniform, caulobasidia 17.2–32.0 × 6.2–8.0 μm, subclavate, with yellowish intracellular pigments. Clamp connections not observed.

Habitat: solitary or scattered on a dark-brown soil of *Quercus mongolica* forest.

Known distribution: currently, only known from Jilin province, China.

Additional collection examined: China. Jilin Province, Jian city, Wunvfeng National Forest Park, under *Quercus mongolica*, on dark-brown soil, alt. 1050 m, 10 August 2019, Yong-Lan Tuo 198 (HMJAU 60201).

Notes: *Butyriboletus subregius* is characterized by a pastel pink pileus, a yellowish green stipe covered with reticulation of the same color and staining blue when the hymenophore and stipe are bruised. Morphologically and phylogenetically, *Bu. subregius* resembles *Bu. autumniregius*, *Bu. primiregius*, *Bu. querciregius*, *Bu. regius* and *Bu. fuscoroseus*. However, *Bu. autumniregius* is distinguished by its autumn fruiting season, a stipe that commonly has dark red stains toward the base, and longer spores with a larger Q value [33]; *Bu. primiregius* is characterized by its late spring season, and a pileus tending to dingy olive-brown as it ages or exposed in sunlight [33]; *Bu. querciregius* differs from *Bu. subregius* in its mycorrhizal host, the dull color of a pileus, relatively longer spores with larger Q value [33]; *Bu. regius* is different from *Bu. subregius* in its pileus covered with distinct scales with aging, a context usually not bluing when cut, and spores longer with larger Q value [69]. *Butyriboletus fuscoroseus* is different from *Bu. subregius* in its brown-pink, reddish brown, or purplish-brown pileus, fine reticulation covered only on the upper half of stipe and context of stipe strongly bluing when cut [56]. Reference Table 2 provides the critical characteristics distinguishing *Bu. subregius* from other species in China.

*Tengioboletus subglutinosus* Yang Wang, Bo Zhang & Yu Li, sp. nov.

Mycobank No.: 842168.

**Figure 9:**
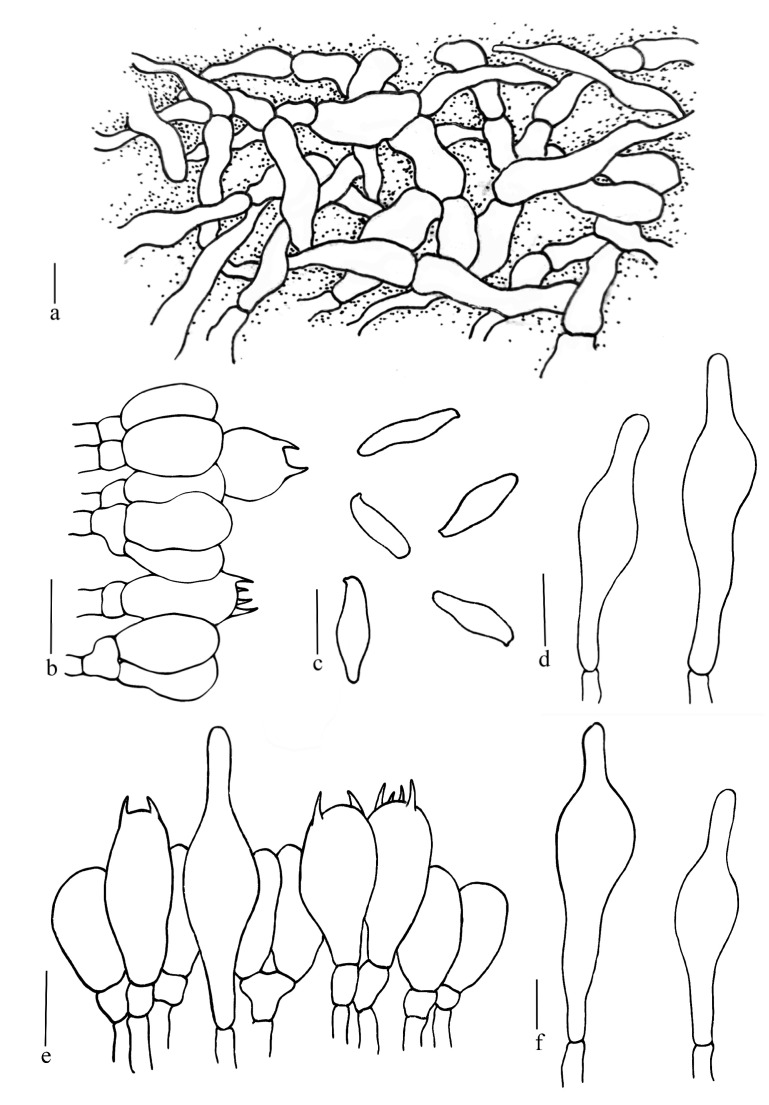
*Tengioboletus subglutinosus*. (**a**) Pileipellis; (**b**) Stipitipellis; (**c**) Basidiospores; (**d**) Cheilocystidia; (**e**,**f**) Pleurocystidia and basidia. Scale bars: 10 μm.

Figure 5f, Figure 7e,f and Figure 9.

Etymology: “sub” means “near,” named because it is similar to *T. glutinosus*.

Holotypus: China. Jilin Province, Jian city, Wunvfeng National Forest Park, 125°34′33″ E, 40°52′7″ N, under *Quercus mongolica*, on dark-brown soil, alt. 650 m, 6 August 2020, Y. L. Tuo 293 (HMJAU 59035!).

Basidiomata medium to large. Pileus 6.5–9.0 cm in diameter, hemispherical to applanate, surface brownish yellow (5C8) to yellowish brown (5D8), glabrous, viscid when wet, context deep yellow (4A8), 0.6–1.5 cm thick, color unchanging when cut; hymenophore sinuate to decurrent; tubes up to 1.3 cm long, vivid yellow (3A8), changing to indistinct blue erratically or unchanging color when cut; hymenophore surface concolorous with tubes or olive yellow (3C8), staining blue when bruised; pores angular, ca. 2–3/mm. Stipe 7.2–16.0 × 1.4–2.2 cm, central, clavate to subcylindrical, solid, sometimes tapered downwards, surface concolorous with pileus surface, covered with fine reticulation at apex, context deep yellow (4A8), color unchanging when cut; basal mycelium yellow (3B8).

Basidiospores (60/2/1) (10.0) 11.5–11.7–11.9 (13.0) × (4.0) 4.2–4.3–4.4 (6.0) μm [Q = (1.70) 2.00–3.17 (3.25) 2.75 ± 0.3], elongate ellipsoid and inequilateral in side view, with distinctly suprahilar depression; greenish yellow (1A8) in 5% KOH, smooth. Hymenophoral trama of the intermediate type between phylloporoid and boletoid types. Basidia 19.0–42.0 × 6.0–13.0 μm, clavate, 2- and 4-spored, hyaline in 5% KOH. Pleurocystidia scattered, 45.0–65.0 × 9.0–15.0 μm, fusoid-ventricose to broadly fusoid-ventricose, with subacute apex or long beak, thin-walled. Cheilocystidia 36.0–50.0 × 7.5–10.5 μm, similar to pleurocystidia in shape. Pileipellis an interwoven ixotrichodermium, with inflated terminal cells 28.5–57.0 × 15.0–23.0 μm. Stipitipellis fertile, hymeniform, with subglobose to globose terminal cells, and scattered clavate basidia.

Habitat: solitary or scattered on a dark-brown soil of *Quercus mongolica* forest.

Known distribution: currently, only known from Jilin province, China.

Additional collections examined: China. Jilin Province, Jian city, Wunvfeng National Forest Park, under *Quercus mongolica*, on dark-brown soil, alt. 900 m, 6 August 2020, Yong-Lan Tuo 286 (HMJAU 59034); alt. 750 m, 11 August 2020, Yong-Lan Tuo 344 (HMJAU 59036); alt. 650 m, 23 August 2020, Yong-Lan Tuo 471 (HMJAU 59037).

Notes: *Tengioboletus subglutinosus* is characterized by a hymenophore surface staining blue when bruised, a pileipellis in the form of an ixotrichodermium, with inflated or clavated terminal cells. Morphologically and phylogenetically, *T. subglutinosus* is similar to *T. glutinosus*. However, *T. subglutinosus* is different due to its hymenophoral surface staining blue when bruised, a hymenophore sinuate to decurrent, a stipe with reticulations at the apex, and narrower spores [5]. *Tengioboletus fujianensis* differs from *T. subglutinosus* in its hymenophoral surface staining brown when bruised, prominently reticulation nearly to the stipe base and hymenophoral trama boletoid [34]. Basidiomata of *T. reticulatus* show a distinct olive-brown, brown-to dark-brown pileus, shorter hymenophore of unchanging color when bruised, a distinct reticulation covering stipe, and a pileipellis trichodermium, not ixotrichodermium [5].

*Suillellus lacrymibasidiatus* Yang Wang, Bo Zhang & Yu Li, sp. nov.

Mycobank No.: 842518.

**Figure 10:**
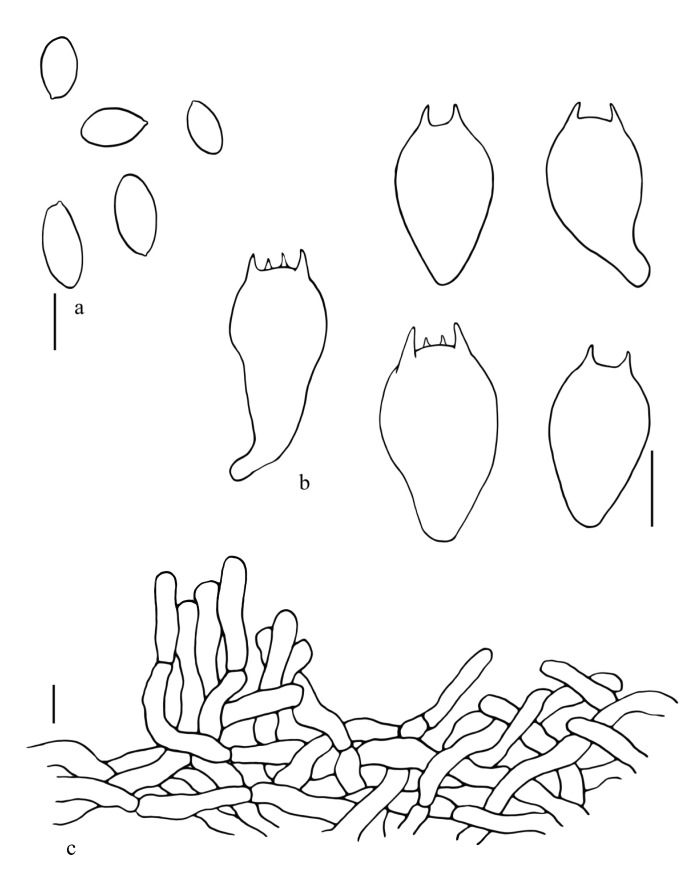
*Suillellus lacrymibasidiatus*. (**a**) Basidiospores; (**b**) Basidia; (**c**) Pileipellis. Scale bars: 10 μm.

Figure 5a–c, Figure 7a,b and Figure 10.

Etymology: “*lacrymibasidiatus*” means most of its basidia seem lacrymoid.

Holotypus: China. Xinjiang Uygur Autonomous Region: Ili Kazakh Autonomous Prefecture, Xinyuan county, 84°31′20″ E, 43°15′43″ N, under *Pinus schrenkiana*, on light grayish brown loess, alt. 1899 m, 3 August 2021, W3194 (HMJAU 60202!).

Basidiomata medium. Pileus 4.1–8.2 cm in diameter, hemispherical then applanate, surface oak brown (5D6) when young, brownish orange (6C6) at maturity, weakly tomentose, context yellowish white (1A2), 0.4–0.9 cm thick, turning blue when cut. Hymenophore adnexed, surface tomato red (8C8) when young, brick red (7D7) at maturity, bluing quickly when injured, pores angular, ca. 1–3/mm; tubes up to 1.3 cm long, sulfur yellow (1A5), bluing promptly when cut. Stipe 7.2–7.4 × 1.7–2.0 cm, subcylindrical, relatively slender at middle part or attenuate downwards, surface red (10A6) when young, finely longitudinally reticulated over the apex, color of surface fading to yellow and covered with distinct squamules at the middle part in ages, context pastel green (30A4), turning blue when cut; basal mycelium white.

Basidiospores (60/2/2) (11.6) 14.5–14.7–15 (17.0) × (6.7) 7.7–7.8–7.9 (9.0) μm, Q = 1.5–2.1, Qm = 1.9 ± 0.1, subamygdaloid to broadly ellipsoid, brown in 5% KOH, smooth, neither amyloid nor dextrinoid. Hymenophoral trama boletoid type, composed of 2.0–16.5 μm wide hyphae, amyloid. Basidia 20.8–38.5 × 13.0–20.1 μm, lacrymoid, 2– and 4–spored, hyaline in 5% KOH. Pleurocystidia and cheilocystidia not observed. Pileipellis a trichodermium, composed of 5.0–9.5 μm wide, yellowish brown, inamyloid hyphae. Stipitipellis hymeniderm, terminal cells inflated, 25.8–61.2 × 12.0–15.5 μm. Hyphae of the flesh in the stipe base amyloid in Melzer’s reagent. Clamp connections not observed.

Habitat: solitary or scattered on a black loam soil of *Salix* spp. and *Populus* spp. mixed forest or a light grayish brown loess of *Pinus schrenkiana* forest.

Known distribution: currently, only known from Xinjiang Uygur Autonomous Region, China.

Additional collection examined: China. Xinjiang Uygur Autonomous Region: Ili Kazakh Autonomous Prefecture, Zhaosu County, 80°42′30″ E, 42°59′37″ N, under river valley with presence of *Salix* spp. and *Populus* spp., on black loam soil, alt. 1697 m, 6 August 2021, W3229 (HMJAU 60203).

Notes: *Suillellus lacrymibasidiatus* is characterized by its oak brown to brownish orange pileus, the context staining blue when injured, and inamyloid basidiospores. Morphologically, *S. lacrymibasidiatus* is related to *S. luridus* (Schaeff.) Murrill, *S. mendax* (Simonini & Vizzini) Vizzini, Simonini & Gelardi, *S. queletii* (Schulzer) Vizzini, Simonini & Gelardi, and *S. subamygdalinus* Kuan Zhao & Zhu L. Yang. *S. luridus* is characterized by its prominent reticulation on the stipe and smaller basidiospores [71]; *S. mendax* is different from *S. lacrymibasidiatus* in its promptly bluing when pileus bruised, value of Q larger, and basidia clavate [13]; *S. queletii* can be distinguished from *S. lacrymibasidiatus* by its stipe wholly covered with fine granulation without reticulation, basidia clavate [72]; *S. subamygdalinus* is characterized by its basidia clavate [5]. Phylogenetically, *S. lacrymibasidiatus* is related to *S. comptus*. However, *S. comptus* differs from *S. lacrymibasidiatus* in its stipe surface staining blue when bruised, and smaller spores [71]. Among the other morphologically allied boletes, *S. adalgisae* (Marsico & Musumeci) N. Schwab [73], *S. austrinus* (Singer) Murrill [74], S. gabretae (Pilát) Blanco-Dios [75], *S. luridiceps* Murrill [76], and *S. subvelutipes* (Peck) Murrill. [77], none of them could represent a possible concurrent of *S. lacrymibasidiatus*.

A key to worldwide species of *Tengioboletus*:
1. Pores changing color when bruised21. Pores unchanging color when bruised32. Pores staining blue when bruised, pileipellis an ixotrichodermium*T. subglutinosus*2. Pores staining brown when bruised, pileipellis an trichodermium*T. fujianensis*3. Stipe covered with distinct reticulations, basidiospores larger, 12.0–14.5 × 4.5–6.0 μm, pileipellis an trichodermium *T. reticulatus*3. Stipe nearly glabrous, basidiospores 10.0–12.0 × 3.5–4.5 μm, pileipellis, an ixotrichodermium*T. glutinosus*

A key to worldwide species of *Suillellus* s.str.:
1. Basidiospores usually longer than 15 μm21. Basidiospores shorter than or equal to 15 μm42. Stipe covered with pruinose or granulose, but without any trace of reticulation*S. amygdalinus*2. Stipe covered with reticulation33. Basidia lacrymoid, Q = 1.5–2.1*S. lacrymibasidiatus*3. Basidia clavate, Q = 2.0–2.6*S. subamygdalinus*4. Surface of stipe rough, but without reticulation54. Stipe covered with reticulation65. Stipe covered with distinct pruinose, basidiospores less than 14 μm, basidia broad clavate*S. adonis*5. Stipe covered with reddish to brown granules, basidiospores can be longer than 14 μm, hymenophoral basidia clavate*S. queletii*6. Stipe covered with prominent, red to orange reticulation. Basidiospores 11–14 × 4.5–6 μm*S. luridus*6. Stipe with indistinctly or finely reticulation, usually distributed erratically77. Q value higher than 2.6*S. mendax*7. Q value less than or equal to 2.688. Basidiospores dextrinoid, Q value can reach 2.6, reticulation yellow and fine at the upper portion of stipe*S. atlanticus*8. Q value less than or equal to 2.2, pores red to orange red, stipe covered with very fine yellow, pale orange, orange, reddish orange, or pale red granules*S. comptus*

## 4. Discussion

In this study, four new species, *Butyriboletus pseudoroseoflavus*, *Butyriboletus subregius*, *Tengioboletus subglutinosus*, and *Suillellus lacrymibasidiatus*, were discovered in northern China based on morphological studies and phylogenetic analyses.

Seven species of *Butyriboletus* were previously reported in China, and all of them were collected from tropical and subtropical regions of China. The two new species of *Butyriboletus* we proposed here are the first reports of this genus in northern China. Moreover, according to Arora et al. [33], the species diversity of the genus should be more abundant in temperate climes than tropical, subtropical, or boreal ones. Based on this, we presume that northern China may be a species diversity hotspot of *Butyriboletus* waiting to be explored further.

*Butyriboletus subregius* is easily confused with *Bu. autumniregius*, *Bu. primiregius*, *Bu. querciregius*, and *Bu. regius*, morphologically. The primary distinguishing characteristics are the fruiting time and different ecological niches. According to Queiroz [78], these differences mean that the features formerly treated as secondary species criteria are relevant to species delimitation, to the extent that they provide evidence of a lineage separation. Although one ITS sequence of *Bu. loyo* (Phillippi) Mikšík, was uploaded to the GeneBank [79], the authors did not give a detailed morphological description to prove identification accuracy, so it was excluded from the current phylogeny. However, *Bu. loyo* is unique within this genus, given its combined morphological characteristics of being equilateral in profile and having red-brown basidiospores and a viscid pileus.

Due to the different color of the hymenophore surface and tubes and the usually vivid red color of basidiomata, Farid et al. [19], Bozok et al. [80], and Biketova et al. [37] all recommended *Exsudoporus* as a genus separate from *Butyriboletus*, including *B. floridanus* (Singer) G. Wu, Kuan Zhao & Zhu L. Yang, *B. frostii* (J.L. Russell) G. Wu, Kuan Zhao, & Zhu L. Yang, and *E. permagnificus* (Pöder) Vizzini, Simonini, & Gelard. However, only the result of Farid et al. [19] showed that the *B. subsplendidus* (W.F. Chiu) Kuan Zhao, G. Wu, & Zhu L. Yang clade has affinity with other *Butyriboletus*. Our phylogram accords with the findings of Chai [28] and Biketova et al. [37] that *B. subsplendidus* is a sister to the *Exsudoporus* clade. We agree with Biketova et al. [37] that *Exsudoporus* should be elevated to genus status, and *B. subsplendidus* and *B. hainanensis* N.K. Zeng, Zhi Q. Liang, & S. Jiang should separate from *Butyriboletus* and represent two distinct genera, as their apparently different characteristics from other species of *Butyriboletus*.

*Tengioboletus* is a small genus, with only three species previously reported in southern China. *Tengioboletus reticulatus* was the first species of the genus collected at Liaoning province in northeastern China [81]. In our study, one new species, *T. subglutinosus*, was also collected at Jilin province in northeastern China. This means a geographical extension of *Tengioboletus* into temperate zones, which may also indicate a potentially wide distribution, given that their previously known main distribution was subtropical and tropical China. Our study showed that sequences of *Tengioboletus* formed an independent clade, which corresponded to the findings of Wu et al. [5] and supported *Tengioboletus* as a separate genus. As found by Wu et al. [5], *Porphyrellus* E.-J. Gilbert is a polyphyletic genus in the phylogram (Figure 1); it formed two clades; one clade named “*Porphyrellus*?” is a sister to *Strobilomyces* Berk., as was implied by Han et al. [82]. Clarification of the relationships among the genera will require additional specimens and future studies.

Recently, many genera were merged or erected in boletes as part of the development of molecular technology. Wu et al. [5] treated *Neoboletus* Gelardi, Simonini, & Vizzini as synonymized with *Sutorius* Halling, Nuhn, & N.A. Fechner, based on molecular data. However, Chai et al. [28] studied the morphological characteristics and reconstructed phylogenetic trees of *Neoboletus*, *Sutorius*, *Costatisporus* T.W. Henkel & M.E. Sm., and *Caloboletus* Vizzini. They considered that *Neoboletus* do not belong to *Sutorius*. Our phylogenetic analyses (Figure 3) confirmed their results.

*Rubroboletus* Kuan Zhao & Zhu L. Yang, *Neoboletus*, *Sutorius*, and *Lanmaoa* G. Wu & Zhu L. Yang shares some characteristics with *Suillellus*, such as the orange-red surface of the hymenophore and the bluing color change. Nevertheless, none of them has the amyloid hyphae of the context [5,25,83,84,85]. In contrast, *Rubroboletus* species have a vivid or dark red pileus with rose-to-red reticulation, and the stipes of species of *Neoboletus* usually have fine dots but never reticulation. The basidiomata of *Sutorius* always have a dull color and a reddish color change [28,86]; the hymenophore of *Lanmaoa* is thin, with a thickness about 1/3–1/5 times that of the pileal context at the position halfway to the pileus center.

## Figures and Tables

**Table 1:** Information of DNA sequences used to reconstruct phylogenetic trees. Sequences newly generated in this study are indicated in bold.

Taxon	Voucher ID	ITS	28S	TEF1	RPB1	RPB2	References
*Tengioboletus glutinosus*	HKAS53425	–	KF112341	KF112204	KF112578	KF112800	[22]
*T. glutinosus*	HKAS53452	–	KT990655	KT990844	KT990994	KT990480	[5]
*T. reticulatus*	HKAS53426	–	KF112491	KF112313	KF112649	KF112828	[22]
*T. reticulatus*	HKAS52241	–	KT990657	KT990845	KT990995	KT990481	[5]
*T. reticulatus*	HKAS53453	–	KT990656	KT990846	–	KT990482	[5]
T. funjianensis	HKAS76661	–	KF112342	KF112205	–	KF112801	[22]
*T. funjianensis*	HKAS77869	–	KT990658	KT990847	KT990996	KT990483	[5]
***T. subglutinosus***	**HMJAU59034 (T286)**	–	**OL588198**	**OL739119**	**OL739121**	–	this study
***T. subglutinosus***	**HMJAU59035 (T293)**	–	**OL588197**	**OL739120**	**OL739122**	**OL739118**	this study
*Porphyrellus porphyrosporus*	MB97-023	–	DQ534643	GU187734	GU187475	GU187800	[50]
*P. porphyrosporus*	HKAS76671	–	KF112482	KF112243	KF112611	KF112718	[22]
*Tylopilus* sp.	HKAS50211	–	KT990552	KT990752	KT990920	KT990389	[22]
*Tylopilus* sp.	HKAS59826	–	KT990558	–	–	–	[5]
*Tylopilus* sp.	HKAS90198	–	KT990559	–	–	–	[5]
*Strobilomyces atrosquamosus*	HKAS55368	–	KT990648	KT990839	KT990989	KT990476	[5]
*S. atrosquamosus*	HKAS78563	–	KT990649	KT990833	KT990983	KT990470	[5]
*S. echinocephalus*	HKAS59420	–	KF112463	KF112256	KF112600	KF112810	[22]
*P.* aff. *alboater*	HKAS55375	–	KT990622	KT990816	KT990969	–	[5]
*P. nigropurpureus*	HKAS74938	–	KF112466	KF112246	–	KF112763	[22]
*P. nigropurpureus*	HKAS52685	–	KT990627	KT990821	KT990973	KT990459	[5]
*P. nigropurpureus*	HKAS53370	–	KT990628	KT990822	KT990974	KT990460	[5]
*P. holophaeus*	HKAS50508	–	KF112465	KF112244	KF112553	–	[22]
*P. holophaeus*	HKAS74894	–	KF112474	KF112245	KF112554	–	[22]
*P. castaneus*	HKAS63076	–	KT990548	KT990749	KT990916	KT990386	[5]
*P. castaneus*	HKAS52554	–	KT990697	KT990883	KT991026	KT990502	[5]
*P. orientifumosipes*	HKAS75078	–	KF112481	KF112242	–	KF112717	[22]
*P. orientifumosipes*	HKAS53372	–	KT990629	KT990823	KT990975	KT990461	[5]
*Boletus bainiugan*	HKAS52235	–	KF112457	KF112203	KF112587	KF112705	[22]
*B. bainiugan*	HKAS55393	–	JN563852	–	JN563868	–	[51]
*B. fagacicola*	HKAS55975	–	JN563853	–	JN563879	–	[51]
*B. fagacicola*	HKAS71347	–	JQ172790	–	JQ172791	–	[51]
*Xanthoconium affine*	NY00815399 (REH8660)	–	KT990661	KT990850	KT990999	KT990486	[5]
*X. porophyllum*	HKAS90217	–	KT990662	KT990851	KT991000	KT990487	[5]
*Baorangia pseudocalopus*	HKAS63607	–	KF112355	KF112167	–	–	[22]
*Ba. pseudocalopus*	HKAS75081	–	KF112356	KF112168	–	–	[22]
*Butyriboletus abieticola*	Arora11087	KC184412	KC184413	–	–	–	[33]
*Bu. appendiculatus*	Bap1	KJ419923	AF456837	JQ327025	–	–	[52]
*Bu. appendiculatus*	BR50200893390-25	KT002598	KT002609	KT002633	–	–	[53]
*Bu. appendiculatus*	BR50200892955-50	KJ605668	KJ605677	KJ619472	–	KP055030	[54]
*Bu. appendiculatus*	MB000286	KT002599	KT002610	KT002634	–	–	[53]
*Bu. autumniregius*	Arora11108	KC184423	KC184424	–	–	–	[33]
*Bu. brunneus*	NY00013631	KT002600	KT002611	KT002635	–	–	[53]
*Bu. fechtneri*	AT2003097	KC584784	KF030270	–	–	–	[21]
*Exsudoporus frostii*	JLF2548	KC812303	KC812304	–	–	–	[33]
*E. frostii*	NY815462	–	JQ924342	KF112164	–	KF112675	[22]
*E. floridanus*	BOS 617, BZ 3170	MN250222	MK601725	MK721079	–	MK766287	[43]
*Bu. hainanensis*	N.K. Zeng 1197 (FHMU 2410)	KU961653	KU961651	–	–	KU961658	[32]
*Bu. hainanensis*	N.K. Zeng 2418 (FHMU 2437)	KU961654	KU961652	KU961656	–	KX453856	[32]
*Bu. huangnianlaii*	N.K. Zeng 3245 (FHMU 2206)	MH885350	MH879688	MH879717	–	MH879740	[28]
*Bu. huangnianlaii*	N.K. Zeng 3246 (FHMU 2207)	MH885351	MH879689	MH879718	–	MH879741	[28]
*Bu. peckii*	3959	–	JQ326999	JQ327026	–	–	[55]
*Bu. persolidus*	Arora11110	KC184444	–	–	–	–	[33]
*Bu. primiregius*	DBB00606	–	KC184451	–	–	–	[33]
*Bu. fuscoroseus*	BR50201618465-02	KT002602	KT002613	KT002637	–	–	[53]
*Bu. fuscoroseus*	BR50201533559-51	KT002603	KT002614	KT002638	–	–	[53]
*Bu. pseudospeciosus*	HKAS59467	–	KF112331	KF112176	–	KF112672	[22]
*Bu. pseudospeciosus*	HKAS63513	–	KT990541	KT990743	–	KT990380	[5]
*Bu. pseudospeciosus*	HKAS63596	–	KT990542	KT990744	–	KT990381	[5]
*Bu. pseudospeciosus*	N.K. Zeng 2127 (FHMU 1391)	MH885349	MH879687	MH879716	–	–	[28]
*Bu. fuscoroseus*	MG383a	KC184458	–	–	–	–	[33]
*Bu. pulchriceps*	DS4514	–	KF030261	KF030409	–	–	[21]
*Bu. pulchriceps*	R. Chapman 0945	KT002604	KT002615	KT002639	–	–	[53]
*Bu. querciregius*	Arora11100	KC184461	–	–	–	–	[33]
*Bu. regius*	MB000287	KT002605	KT002616	KT002640	–	–	[53]
*Bu. regius*	MG408a	KC584789	KC584790	–	–	–	[33]
*Bu. regius*	PRM:923465	KJ419920	KJ419931	–	–	–	[56]
*Bu. roseoflavus*	Arora11054	KC184434	KC184435	–	–	–	[33]
*Bu. roseoflavus*	HKAS63593	KJ909517	KJ184559	KJ184571	–	–	[53]
*Bu. roseoflavus*	HKAS54099	KJ909519	KF739665	KF739779	–	–	[53]
*Bu. roseoflavus*	N.K. Zeng 2123 (FHMU 1387)	MH885348	MH879686	MH879715	–	–	[28]
***Bu. pseudoroseoflavus***	**HMJAU59470 (T274)**	**OL604164**	**OL587853**	**OL739124**	–	**OL739126**	this study
***Bu. pseudoroseoflavus***	**HMJAU59471 (R383)**	**OL604165**	**OL587852**	**OL739123**	–	**OL739125**	this study
*Bu. Roseopurpureus*	E.E. Both3765	KT002606	KT002617	KT002641	–	–	[53]
*Bu. Roseopurpureus*	JLF2566	KC184466	KC184467	–	–	–	[33]
*Bu. Roseopurpureus*	MB06-059	KC184464	KF030262	KF030410	–	–	[21]
*Bu. sanicibus*	Arora99211	KC184469	KC184470	–	–	–	[33]
***Bu. subregius***	**HMJAU60200 (T95)**	**OM237336**	**OM237339**	**OM285111**	–	**OM285109**	this study
***Bu. subregius***	**HMJAU60201 (T198)**	**OM237337**	**OM237340**	**OM285112**	–	**OM285110**	this study
*Butyriboletus* sp.	MHHNU7456	–	KT990539	KT990741	–	KT990378	[5]
*Butyriboletus* sp.	HKAS52525	–	KF112337	KF112163	–	KF112671	[22]
*Butyriboletus* sp.	HKAS57774	–	KF112330	KF112155	–	KF112670	[22]
*Bu. hainanensis*	HKAS59814	–	KF112336	KF112199	–	KF112699	[22]
*Butyriboletus yicibus*	HKAS63528	–	KF112332	KF112156	–	KF112673	[22]
*Bu. Subappendiculatus*	MB000260	KT002607	KT002618	KT002642	–	–	[53]
*Bu. subsplendidus*	HKAS52661	–	KF112339	KF112169	–	KF112676	[5]
*Bu. taughannockensis*	263101	MH257559	MH236172	–	–	–	
*Bu. taughannockensis*	250839	MH234472	MH234473	–	–	–	
*Bu. taughannockensis*	252208	MH236100	MH236099	–	–	–	
*Bu. yicibus*	Arora9727	KC184474	KC184475	–	–	–	[33]
*Bu. yicibus*	HKAS57503	KT002608	KT002620	KT002644	–	–	[53]
*Bu. yicibus*	HKAS68010	KJ909521	KT002619	KT002643	–	–	[53]
*Gymnogaster boletoides*	NY01194009 (REH9455)	–	KT990572	KT990768	–	KT990406	[5]
*Rugiboletus brunneiporus*	HKAS83209	–	KM605134	KM605144	–	KM605168	[27]
*R. extremiorientalis*	HKAS63635	–	KF112403	KF112198	–	KF112720	[22]
*Crocinoboletus laetissimus*	HKAS50232	—	KT990567	KT990762	KT990925	—	[5]
*C. laetissimus*	HKAS59701	—	KF112436	—	—	KF112711	[22]
*C. rufoaureus*	HKAS53424	—	KF112435	KF112206	KF112533	KF112710	[22]
*C. rufoaureus*	HKAS59820	—	KF112434	—	KF112532	KF112709	[22]
*Cyanoboletus brunneoruber*	HKAS63504	—	KF112368	KF112194	KF112531	KF112702	[22]
*Cy. brunneoruber*	HKAS80579 (1)	—	KT990568	KT990763	KT990926	KT990401	[5]
*Cy. brunneoruber*	HKAS80579 (2)	—	KT990569	KT990764	KT990927	KT990402	[5]
*Cy. instabilis*	HKAS59554	—	KF112412	KF112186	KF112528	KF112698	[22]
*Cy. pulverulentus*	9606	—	KF030313	KF030418	KF030364	—	[21]
*Baorangia pseudocalopus*	HKAS63607	—	KF112355	KF112167	KF112519	KF112677	[22]
*Ba. pseudocalopus*	HKAS75081	—	KF112356	KF112168	KF112520	KF112678	[22]
*Lanmaoa angustispora*	HKAS74765	—	KF112322	KF112159	KF112521	KF112680	[22]
*L. angustispora*	HKAS74752	—	KM605139	KM605154	KM605166	KM605177	[27]
*L. angustispora*	HKAS74759	—	KM605140	KM605155	KM605167	KM605178	[27]
*L. asiatica*	HKAS54094	—	KF112353	KF112161	KF112522	KF112682	[22]
*L. asiatica*	HKAS63516	—	KT990584	KT990780	KT990935	KT990419	[5]
*L. fragrans*	18555	JF907800	–	–	–	–	
*Neoboletus brunneissimus*	HKAS52660	—	KF112314	KF112143	KF112492	KF112650	[22]
*N. hainanaensis*	HKAS63515	—	KT990614	KT990808	KT990964	KT990449	[5]
*N. ferrugineus*	HKAS77617	—	KT990595	KT990788	KT990943	KT990430	[5]
*N. ferrugineus*	HKAS77718	—	KT990596	KT990789	KT990944	KT990431	[5]
*N. flavidus*	HKAS59443	—	KU974139	KU974136	KU974142	KU974144	[5]
*N. flavidus*	HKAS58724	—	KU974140	KU974137	KU974143	KU974145	[5]
*Porphyrellus castaneus*	HKAS52554	—	KT990697	KT990883	KT991026	KT990502	[5]
*P. castaneus*	HKAS63076	—	KT990548	KT990749	KT990916	KT990386	[5]
*P. castaneus*	HKAS68575	—	KT990560	—	—	—	[5]
*P. holophaeus*	HKAS59407	—	KT990708	KT990888	KT991030	KT990506	[5]
*P. nigropurpureus*	HKAS52685	—	KT990627	KT990821	KT990973	KT990459	[5]
*P. nigropurpureus*	HKAS53370	—	KT990628	KT990822	KT990974	KT990460	[5]
*P. orientifumosipes*	HKAS75078	—	KF112481	KF112242	—	KF112717	[22]
*P. orientifumosipes*	HKAS53372	—	KT990629	KT990823	KT990975	KT990461	[5]
*Rubroboletus dupainii*	JAM0607	—	—	KF030413	KF030361	—	[21]
*R. latisporus*	HKAS63517	—	KP055022	KP055019	KP055025	KP055028	[25]
*R. latisporus*	HKAS80358	—	KP055023	KP055020	KP055026	KP055029	[25]
*R. rhodosanguineus*	4252	—	KF030252	KF030412	—	—	[21]
*R. rhodoxanthus*	HKAS84879	—	KT990637	KT990831	KT990981	KT990468	[5]
*R. sinicus*	HKAS68620	—	KF112319	KF112146	KF112504	KF112661	[22]
*R. sinicus*	HKAS56304	—	KJ605673	KJ619483	KJ619482	KP055031	[54]
*Suillellus amygdalinus*	112605ba	—	JQ326996	JQ327024	KF030360	—	[55]
*S. amygdalinus*	NY00035656 (Thiers54483)	—	KT990650	KT990840	KT990990	KT990477	[5]
*S. subamygdalinus*	HKAS57262	—	KF112316	KF112174	KF112501	KF112660	[22]
*S. subamygdalinus*	HKAS53641	—	KT990651	KT990841	KT990991	KT990478	[5]
*S. subamygdalinus*	HKAS57953	—	KT990652	KT990842	KT990992	—	[5]
*S. subamygdalinus*	HKAS74745	—	KT990653	KT990843	KT990993	KT990479	[5]
***S. lacrymibasidiatus***	**HMJAU60202 (W3194)**	**OM237315**	**OM230174**	**OM285117**	**OM285113**	**OM285115**	this study
***S. lacrymibasidiatus***	**HMJAU60203 (W3229)**	**OM237338**	**OM230172**	**OM285116**	–	**OM285114**	this study
*Sutorius eximius*	REH9400	—	JQ327004	JQ327029	—	—	[55]
*Su. eximius*	HKAS52672	—	KF112399	KF112207	KF112584	KF112802	[22]
*Su. luridiformis*	AT1998054	UDB000658	–	–	–	–	
*Tylopilus alpinus*	HKAS55438	—	KF112404	KF112191	KF112538	KF112687	[22]
*Ty. argillaceus*	HKAS90201	—	KT990588	KT990783	—	—	[5]
*Ty. argillaceus*	HKAS90186	—	KT990589	KT990784	—	KT990424	[5]
*Ty. atripurpureus*	HKAS50208	—	KF112472	KF112283	KF112620	KF112799	[22]
*Ty. badiceps*	MB03-052	—	KF030336	—	—	—	[21]
*Ty. badiceps*	78206	—	KF030335	KF030429	—	—	[21]
*Veloporphyrellus alpinus*	HKAS68301	—	JX984538	JX984550	—	—	[57]
*V. alpinus*	HKAS57490	—	KF112380	KF112209	KF112555	KF112733	[22]
*V. conicus*	BZ2408	—	JX984545	—	—	—	[57]
*V. conicus*	BZ1670	—	JX984543	JX984555	—	—	[57]
*V. gracilioides*	HKAS53590	—	KF112381	KF112210	KF112556	KF112734	[22]
*Zangia citrina*	HKAS52677	—	HQ326940	HQ32687	—	—	[58]
*Z. citrina*	HKAS52684	—	HQ326941	HQ326872	—	—	[58]
*Z. erythrocephala*	HKAS52843	—	HQ326943	—	—	—	[58]
*Z. erythrocephala*	HKAS52844	—	HQ326944	—	—	—	[58]
*Z. olivaceobrunnea*	HKAS52275	—	HQ326947	HQ326875	—	—	[58]
*Z. roseola*	HKAS75046	—	KF112414	KF112269	KF112579	KF112791	[22]
*S. luridus*	IB2004270	EF644104	–	–	–	–	[59]
*S. luridus*	18902	JF907802	–	–	–	–	[60]
*S. luridus*	18182	JF907793	–	–	–	–	[60]
*S. luridus*	Blu3	AY278765	–	–	–	–	[61]
*S. luridus*	AMB12636	KC734542	–	–	–	–	[13]
*S. luridus*	AMB12638	KC734544	–	–	–	–	[13]
*S. luridus*	TL-6877	AJ889930	–	–	–	–	
*S. luridus*	TL-6877	UDB000077	–	–	–	–	
*S. luridus*	1968	AY278766	–	–	–	–	[61]
*S. luridus*	BL2-VII-10	JQ685714	–	–	–	–	[61]
*S. luridus*	AT-04	UDB002401	–	–	–	–	
*S. luridus*	UP12	DQ658866	–	–	–	–	[62]
*S. luridus*	17696	JF907789	–	–	–	–	[60]
*S. luridus*	BL1-VII-09	JQ685715	–	–	–	–	
*S. luridus*	MA-Fungi 47706	AJ419191	–	–	–	–	[63]
*S. mendax*	AMB12632	KC734547	–	–	–	–	[13]
*S. mendax*	AMB12633	KC734548	–	–	–	–	[13]
*S. mendax*	AMB12634	KC734543	–	–	–	–	[13]
*S. mendax*	AMB12635	KC734545	–	–	–	–	[13]
*S. mendax*	AMB12637	KC734540	–	–	–	–	[13]
*S. mendax*	AMB12640	KC734541	–	–	–	–	[13]
*Boletus luridus*	UF107	HM347662	–	–	–	–	
*B. amygdalinus*	src491	DQ974705	–	–	–	–	[64]
*B. comptus*	17827	JF907791	–	–	–	–	[60]
*B. comptus*	AMB12639	KC734539	–	–	–	–	[13]
*B. queletii*	17196	JF907784	–	–	–	–	[60]
*B. queletii*	17208	JF907785	–	–	–	–	[60]
*B. queletii*	AMB12641	KC734546	–	–	–	–	[13]
*B. queletii*	JV01-231	UDB000760	–	–	–	–	
*N. erythropus*	MA-Fungi 47702	AJ419188	–	–	–	–	[63]
*N. erythropus*	BOER_TO_2 (AAM630/06)	FM958177	–	–	–	–	
*N. erythropus*	UF278	HM347644	–	–	–	–	
*N. erythropus*	UF276	HM347643	–	–	–	–	
*N. erythropus*	UF269	HM347665	–	–	–	–	
*N. erythropus*	DG05-54	UDB001523	–	–	–	–	
*N. erythropus*	SU46	DQ131633	–	–	–	–	[65]
*N. erythropus*	SU47	DQ131634	–	–	–	–	[65]
*N. erythropus*	Daniels 582	AJ496595	–	–	–	–	[63]
*Caloboletus calopus*	AT1998059	UDB000659	–	–	–	–	
*Ca. radicans*	TUF106003	UDB003224	–	–	–	–	
*Bu. fuscoroseus*	AH96025	UDB000649	–	–	–	–	
*Bu. fuscoroseus*	AT1996017	UDB000652	–	–	–	–	
*Bu. fechtneri*	AT2003097	UDB000703	–	–	–	–	[21]
*Imperator rhodopurpureus*	AT1996058	UDB000654	–	–	–	–	
*R. pulchrotinctus*	GS0860	UDB000407	–	–	–	–	
*R. satanas*	AT1998051	UDB000415	–	–	–	–	
*R. rubrosanguineus*	GS0405	UDB000410	–	–	–	–	
*R. rhodoxanthus*	AT2000182	UDB001116	–	–	–	–	
*Cyanoboletus pulverulentus*	RT00004	EU819502	–	–	–	–	
*Cyanoboletus pulverulentus*	AH97030	UDB000408	–	–	–	–	[66]
*B. aestivalis*	AT2004040	UDB001113	–	–	–	–	
*B. aereus*	AT2000198	UDB000943	–	–	–	–	

**Table 2:** Morphological comparisons of *Butyriboletus pseudoroseoflavus* sp. nov. and *Butyriboletus subregius* sp. nov. with other *Butyriboletus* spp. reported in China.

Species	Pileus	Context	Hymenophore	Stipe	Spores	Cystidia
*Butyriboletus huangnianlaii*	Surface dry, finely tomentose, brown to reddish brown	Yellowish to yellow, changing blue quickly when injured	Adnate or slightly depressed, changing blue quickly when injured	Stipitipellis, fertile hymeniform, fusiform, or subfusiform terminal cells	(7.0) 7.5–10.5 (11.0) × 3.0–4.0 μm, olive-brown to yellowish brown	Fusiform or subfusiform
*Bu. pseudospeciosus*	Purplish tint, staining dark blue quickly when bruised	Yellowish, staining blue to grayish blue promptly when injured	Adnate, rapidly bluing when bruised	Stipitipellis consisting of tufts of lageniform caulocystidia	9.0–11.0 (12.0) × 3.5–4.0 μm	Narrowly lageniform to lageniform
*Bu. roseoflavus*	Pinkish to purplish red or rose-red	Yellowish or light yellow, turning blue slowly or unchanging when bruised	Adnate, staining blue quickly when hurt	Stipe trama composed of parallel hyphae	9.0–12.0 (13.0) × 3.0–4.0 μm	Narrowly lageniform to lageniform
*Bu. sanicibus*	Dull brown	Pale yellow, usually turning blue when cut	Depressed, bruising blue	–	11.0–15.0 × 4.0–5.0 μm	Fusoid-ventricose
*Bu. subregius*	Pastel pink	Yellowish green, turning blue when cut	weakly decurrent, covered with a layer of whitish mycelium when young, surface yellowish green	Stipitipellis fertile, hymeniform, caulocystidia narrowly lageniform, caulobasidia subclavate, with yellowish intracellular pigments.	(10.0) 11.1–11.5 (13.0) × (3.0) 4.0–4.2 (5.0) μm	narrowly lageniform
*Bu. yicibus*	Covered with fibrillose squamules, ochreous, brown to dark brown	Nearly white, staining light blue very slowly when injured	Adnate, degrading bluish slowly when injured	Stipitipellis consisting of tufts of lageniform caulocystidia	(11.0) 13.0–15.0 (16.0) × 4.0–5.0 (5.5) μm	Narrowly lageniform to lageniform
*Bu. pseudoroseoflavus*	Tomentose, pink to greyish rose	Light yellow, unchanging in color when injured.	Adnate to decurrent, staining blue when bruised	Stipitipellis hymeniform, with terminal inflated cells	(7.0) 10.2–11.0 (16.0) × (2.0) 3.1–3.7 (4.0) μm	Narrowly lageniform

## Data Availability

Data relevant to this research can be found here: https://www.ncbi.nlm.nih.gov/; https://www.mycobank.org/; https://www.treebase.org/treebase-web/home.html, accessed on 15 January 2022.

## Supplementary Materials

The following are available online at https://www.mdpi.com/article/10.3390/jof8030218/s1, File S1: *Tengioboletus* matrix, File S2: *Butyriboletus* matrix, File S3: *Suillellus* multigene matrix, File S4: *Suillellus* ITS matrix.
